# L‐type Voltage‐Gated calcium channels partly mediate Mechanotransduction in the intervertebral disc

**DOI:** 10.1002/jsp2.1213

**Published:** 2022-06-23

**Authors:** Philip Poillot, Joseph W. Snuggs, Christine L. Le Maitre, Jacques M. Huyghe

**Affiliations:** ^1^ Bernal Institute University of Limerick Limerick Ireland; ^2^ Biomolecular Sciences Research Centre Sheffield Hallam University Sheffield UK; ^3^ Department of Mechanical Engineering Eindhoven University of Technology Eindhoven The Netherlands

**Keywords:** calcium, degeneration, intervertebral disc, mechanotransduction, voltage gated ion channel

## Abstract

**Background:**

Intervertebral disc (IVD) degeneration continues to be a major global health challenge, with strong links to lower back pain, while the pathogenesis of this disease is poorly understood. In cartilage, much more is known about mechanotransduction pathways involving the strain‐generated potential (SGP) and function of voltage‐gated ion channels (VGICs) in health and disease. This evidence implicates a similar important role for VGICs in IVD matrix turnover. However, the field of VGICs, and to a lesser extent the SGP, remains unexplored in the IVD.

**Methods:**

A two‐step process was utilized to investigate the role of VGICs in the IVD. First, immunohistochemical staining was used to identify and localize several different VGICs in bovine and human IVDs. Second, a pilot study was conducted on the function of L‐type voltage gated calcium channels (VGCCs) by inhibiting these channels with nifedipine (Nf) and measuring calcium influx in monolayer or gene expression from 3D cell‐embedded alginate constructs subject to dynamic compression.

**Results:**

Several VGICs were identified at the protein level, one of which, Cav2.2, appears to be upregulated with the onset of human IVD degeneration. Inhibiting L‐type VGCCs with Nf supplementation led to an altered cell calcium influx in response to osmotic loading as well as downregulation of col 1a, aggrecan and ADAMTS‐4 during dynamic compression.

**Conclusions:**

This study demonstrates the presence of several VGICs in the IVD, with evidence supporting a role for L‐type VGCCs in mechanotransduction. These findings highlight the importance of future detailed studies in this area to fully elucidate IVD mechanotransduction pathways and better inform treatment strategies for IVD degeneration.

## Graphical abstract

This study investigated the expression and function of voltage‐gated ion channels in the intervertebral disc. One channel, Cav2.2, was found to be upregulated in degeneration, while L‐type voltage gated calcium channels were found to be involved in mediating calcium signaling and the response of disc cells to dynamic compression.

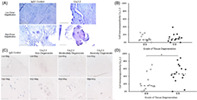

## 1 INTRODUCTION

The intervertebral disc (IVD) is a load‐bearing cartilaginous joint in the spine, composed of two distinct tissues. The inner nucleus pulposus (NP) is a highly hydrated tissue composed primarily of proteoglycans (PG) and types I and II collagen, while the outer annulus fibrosus (AF) is a fibrous ring, with a lower concentration of PGs and higher proportion of collagen oriented in lamellae.^1^ Such a structure allows the IVD to sustain loads by balancing hydrostatic pressure in the NP with constraining tensile forces in the AF,^2^ all the while inducing variable levels of compression, torsion, and fluid‐induced shear stresses.

However, through IVD degeneration, the composition and structure of these tissues undergo dramatic changes. With strong links to lower back pain,^3^ IVD degeneration is a widespread and growing global health problem^4^ which can alter normal spine biomechanics and lead to complex and uneven loading patterns. As less PGs are synthesized by the native cells and increased degradation, the IVD starts to lose its water content, while collagen type changes, becomes denatured and cross linking changes as it invades the NP.^5^ This leads to a loss of osmotic pressure and disc height and fissure formation throughout the disc.^1^ Cells can also cluster and change phenotype, producing more cytokines and proteinases,^6^, ^7^ which are linked to an altered extracellular matrix (ECM).^8^ These degenerative changes then alter the physiological forces acting through the disc under load, whereby less hydrostatic pressure is generated in the NP, leading to increased shear stress and stress concentrations through the IVD.^9^

Physiological loading of the IVD generates a strain‐generated potential (SGP), comprised of Donnan equilibrium potentials and electrokinetic potentials.^10^, ^11^, ^12^ Electrokinetic phenomena, such as streaming and diffusion potentials, are generated through fluid‐driven flow around the negatively charged ECM of native PGs.^13^ While these only persist as fluid and ion flow occur, Donnan potentials resulting from fixed charge differences remain. The SGP has been recorded in the IVD^14^ but has been studied more closely in the similar tissue of cartilage. Chondrocytes are known to respond to a variety of mechanical forces, such as compression,^15^ tension,^16^ and shear stress^17^ through voltage‐gated ion channels (VGICs). These VGICs activate in response to a change in voltage across the plasma membrane, whereby positively charged helices in the voltage‐sensing domain are attracted to the negatively charged side of the membrane.^18^ This causes the pore complex of the channel to open or close, allowing for ion flux specific to that channel, such as calcium or sodium. As the IVD generates a SGP similar to cartilage, this implicates VGICs in the mechanotransduction pathway in the disc.

Cells in the IVD are known to respond to a variety of forces through many different mechanoreceptors, such as integrins,^19^, ^20^ transient receptor potential (TRP) ion channels,^21^, ^22^ cytoskeletal remodeling,^23^ and endoplasmic reticulum stress.^24^ However, little is known about how the IVD responds specifically to the SGP. Only genomic microarray data from mice has suggested the expression of VGICs in the IVD.^25^ Even less is known how cells in a degenerate IVD may respond to an altered SGP through altered loading patterns. Some signaling pathways have been shown to be altered in IVD degeneration, such as the integrin‐mediated response to dynamic compression,^20^ highlighting the necessity to conduct such mechanotransduction studies in both the healthy and degenerated IVDs.

The objective of this study is to investigate the presence and function of VGICs in the IVD. In doing this, the study is split into two distinct parts. Firstly, the expression of several VGICs are investigated for the first time in the disc. Leveraging microarray data,^25^ four VGIC markers that were expressed at the gene level in mice were chosen for investigation at the protein level in human and bovine IVD: Ca_v_2.2, a voltage‐gated calcium channel (VGCC); Na_v_1.1, a voltage‐gated sodium channel; K_v_3.3, a voltage‐gated potassium channel; and CACNA2D1, an auxiliary subunit of several different voltage‐gated calcium channels. Given the evidence of L‐type VGCCs in chondrocytes,^15^, ^16^, ^26^ the expression of one such channel, Ca_v_1.2, was also investigated in the human IVD. The expression of these channels was further investigated across different grades of degeneration in the human NP.

**FIGURE 1:**
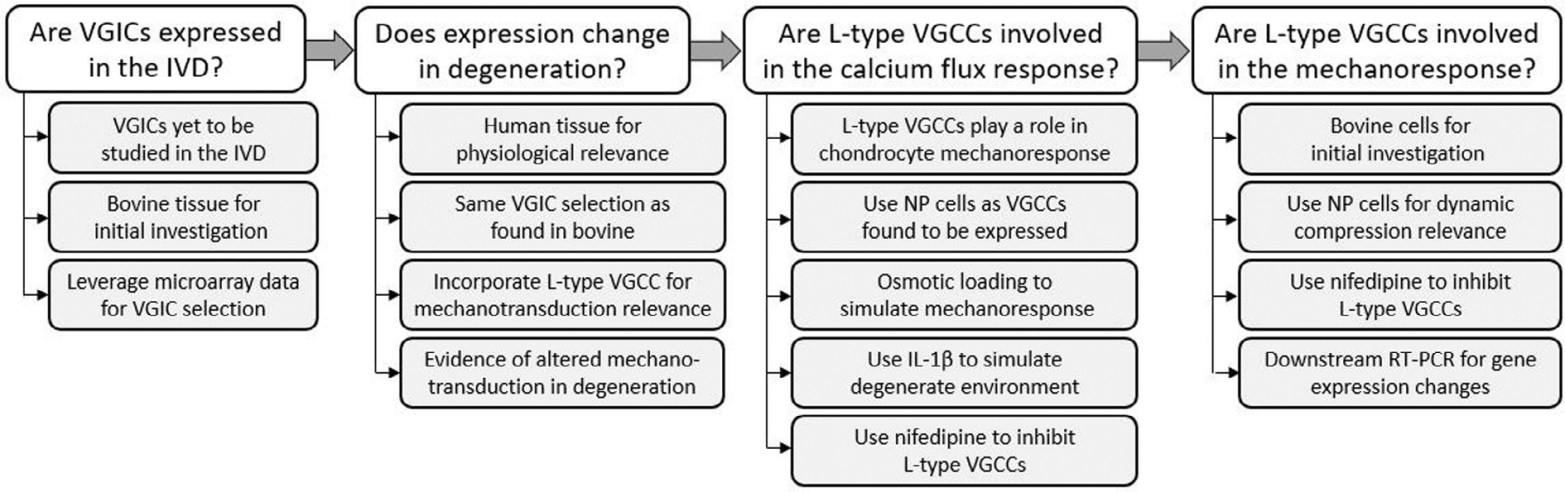
Flow diagram describing the sequential research questions addressed in this study and the associated rationale for the investigations

In the second part of this study, the function of VGICs in the IVD was investigated. L‐type VGCCs were chosen for functional investigation due to their demonstrated roles in chondrocyte mechanotransduction.^15^, ^16^, ^26^ In doing so, calcium signaling of IVD cells in response to osmotic loading was first measured with/without nifedipine (Nf) supplementation, a commonly used inhibitor of L‐type VGCCs, to allow for comparison of results and determination of the proportion of response mediated by L‐type VGCCs. As mechanotransduction and signaling pathways can be altered in degeneration,^20^, ^27^ calcium signaling was also investigated in the presence of IL‐1β (IL‐1) supplementation, a cytokine that has been shown to be an important component of disc degeneration.^28^, ^29^, ^30^ Following this, 3D dynamic compression and downstream gene analysis was conducted in the presence of the same inhibitor to further examine the role of VGCCs in IVD mechanotransduction. While gene analysis was performed for anabolic, catabolic, and VGIC markers, PTHrP expression was also incorporated as it is increased in degeneration^31^ while being shown to be regulated by VGICs in the chondrocyte mechanoresponse.^16^ This two‐step approach, with the rationale for the research questions outlined in Figure 1, was taken to best inform future studies on VGICs and the SGP in the IVD.

## 2 MATERIALS AND METHODS

### 2.1 Bovine tissue

Bovine IVD tissue, harvested from bovine tails, was obtained from 18–24 month old animals from a local abattoir and processed within 2 h of slaughter. IVD tissue from caudal levels 1, 2, and 3 were isolated by scalpel, while AF and NP segments were further separated prior to tissue processing. AF and NP segments were fixed in 4% paraformaldehyde for 48 h at 4°C to preserve the tissue. Following fixation, segments were immersed in 30% sucrose solution for 24 h at 4°C to protect the tissue structure during the subsequent freezing process. Tissue was snap frozen by immersion in liquid nitrogen. Frozen segments were stored at −80°C until sectioning (<2 months) to 15 μm‐thick sections on Superfrost Plus glass slides (VWR) and stored at −80°C prior to immunofluorescent staining (<2 weeks).

### 2.2 Human tissue

Human IVD tissue was obtained from patients through surgical intervention or from cadaveric sources with informed consent of the patients or relatives and ethical approval from Sheffield Research Ethics Committee (09/H1308/70). Samples from 42 different patients (age: 48 ± 30 years old, sex: 21 female/19 male/2 unknown, IVD level: 31 lumbar/6 cervical/4 unknown) obtained by surgical intervention (discectomy/corpectomy/decompression 84%) or post‐mortem examination (16%) were selected for immunohistochemical (IHC) staining representing a range of histological grades. Tissue was fixed in 10% v/v neutral buffered formalin (Leica Microsystems) and embedded into paraffin wax before being cut to 4 μm sections and mounted to positively charged slides (Leica). As many tissue samples only included NP tissue, this region was utilized for grading using the newly published consensus grading scheme for human IVD tissues.^32^

### 2.3 Fluorescent immunohistochemistry

Fluorescent IHC was performed on bovine AF and NP tissue sections to investigate the presence of VGICs. Staining for the four VGIC markers was performed using polyclonal primary antibodies from Atlas Antibodies (Table 1). An isotype control for rabbit IgG (AB‐105‐C; Bio‐Techne) was also included in both AF and NP sample slides at equivalent IgG concentration to primary antibodies. Following preliminary staining, all four targets appeared present in AF while only the Ca_v_2.2 target appeared present in NP tissue. Thus, sections of AF and NP from at least 6 IVDs (across three different animals) were stained for Ca_v_2.2, while similar staining of only AF sections was performed for Na_v_1.1, K_v_3.3 and CACNA2D1. IHC was performed as described previously.^33^ Sections were incubated with goat anti‐rabbit FITC secondary antibody (31 635; Invitrogen) and counterstained with DAPI (D9542; Sigma Aldrich) before mounting with Fluoromount (Sigma Aldrich) and stored at 4°C until imaging (<2 weeks). Imaging was performed using a confocal microscope (Zeiss) with a 20x objective through both the 405 nm (for DAPI) and 488 nm (for targets) wavelengths to quantify the number of cells expressing the target channels of interest. At least 100 cells were counted in each section for positive or negative staining of the antibody of interest. Images were similarly taken using the 63× objective to localize the expression of those channel proteins across the cell, as well as in z‐stack increments of 1.5 μm.

**TABLE 1:** Primary antibodies used in immunohistochemical (IHC) staining of voltage‐gated ion channels in bovine and human intervertebral disc (IVD)

Antibody target	Catalogue number	Channel	Channel family	Dilution
CACNA1B	HPA044347	Ca_v_2.2	N‐type voltage‐gated calcium channel	1:50
CACNA2D1	HPA008213	Auxiliary subunit	Several voltage‐gated calcium channels	1:50
KCNC3	HPA018041	K_v_3.3	Voltage‐gated potassium channel	1:100
SCN1A	HPA078664	Na_v_1.1	Voltage‐gated sodium channel	1:20

### 2.4 Immunohistochemistry (colorimetric)

IHC was performed on paraffin‐embedded disc sections to quantify the expression of VGICs in the human disc. Primary antibodies, as well as the IgG isotype control, were the same of those used for immunoflourescent staining (Table 1). In addition, expression of the Ca_v_1.2 channel, an L‐type VGCC, was also investigated as L‐type VGCCs were the subsequent focus of this study due to the role this family of channel likely plays in mechanotransduction. Ca_v_1.2 was stained using a monoclonal primary antibody raised in mouse (ab84814;Abcam) and an IgG1 control (sc‐3877; Santa Cruz Biotechnology). While all markers were tested across a range of methods, the optimum antigen retrieval method and dilution ratios were found to be heat retrieval and 1:200/1:250 for Ca_v_1.2 and Ca_v_2.2 respectively. IHC staining for Ca_v_2.2 was performed utilizing the recommended IHC methodology as described by Reference [34]. Briefly, endogenous peroxidises were blocked by hydrogen peroxide in ethanol, antigen retrieval was performed by heating in tris buffer and non‐specific protein interaction was blocked by goat serum. Sections were incubated with primary antibodies in TBS at 4 °C O/N before incubation with secondary antibodies. ABC solution (Vector Labs) and DAB solution (Sigma Aldrich) were applied before counterstaining with hematoxylin and mounting with Pertex. Staining for Ca_v_1.2 was performed in a similar method using the ImmPRESS HRP Universal PLUS Polymer kit (MP‐7800; Vector Labs) without ABC solution and mounted in DPX. At least 100 cells, or all cells present, were counted in each section for positive or negative staining of each antibody. Percentage cells with immunopositive staining were calculated and patient discs graded 0–4 (Non/Low degeneration) v/s 5–9 (Medium/Severe Degeneration) were statistically analyzed (see below).

### 2.5 Calcium signaling

The function of VGICs, particularly VGCCs, in the IVD was first investigated through monolayer cell stimulation and measurement of the resultant cell calcium influx by the Fluo‐4 direct calcium assay with/without supplementation of nifedipine, an inhibitor of L‐type VGCCs. While Cav2.2 was found to be differentially expressed in degeneration, which is an N‐type VGCC, the L‐type VGCC family, which includes Cav1.2, was investigated for their roles in mechanotransduction due to the evidence for this function in chondrocytes, building an initial evidence base for VGCCs in the IVD. Human NP cells were isolated from surgically removed NP tissue of three different patients as described previously^35^ and cultured in monolayer (<P2) in standard culture media (High‐glucose DMEM (Gibco) containing 10 000 U/mL pen/strep, 50 μg/mL amphotericin B, 50 μg/mL ascorbic acid, and 10% FBS (v/v)). Bovine NP cells were similarly extracted from bovine tails. Cells were then seeded in a 96‐well plate at 10 000 cells/well with pre‐treatments IL‐1, Nf or IL‐1 + Nf for 24 h. A concentration of 10 ng/mL IL‐1 (Peprotech) was used as previously demonstrated,^28^, ^30^, ^36^ while 1 mM Nf was chosen as it is one of the lower working concentrations that have been verified and widely used to inhibit L‐type VGCCs in chondrocytes.^15^, ^16^, ^37^, ^38^, ^39^ Calcium influx was investigated using the Fluo‐4 Direct Calcium Assay kit (F10471; Invitrogen) following the manufacturer's instructions and as described previously.^22^ Briefly, Fluo‐4 was added to each well of the 96‐well plate after 24 h culture in pre‐treatment media. After 30 min incubation, plates were inserted into a microplate reader for stimulation and subsequent calcium influx measurement by reading fluorescence excitation at 488 nm. Measurements were taken from cells of three different patients seeded each into three different wells for each condition, while bovine cells were tested in six wells for each condition. Each well was first measured for baseline intracellular calcium levels for 5 s with readings at 50 ms intervals. Wells were then injected with treatments, after which the well was again measured for 50 s at 50 ms intervals. Treatments included standard media (approx. 325 mOsm) with/without IL‐1, Nf or both, as well as each condition in osmotically altered media (approx. 225 mOsm after injection) by supplementation with sterile dH_2_O at 38.5% (v/v). This hypo‐osmotic media was used to represent physiological changes in the IVD during degeneration, or the removal of mechanical loading in diurnal cycles.^22^, ^40^ Excitation measurements following injection were normalized to baseline pre‐injection to account for changes in media volume. The magnitude of peak fluorescent intensity and the time taken to reach peak fluorescence intensity (in relation to start of baseline reading) were recorded for each well.

### 2.6 Mechanotransduction study

#### 2.6.1 3D cell culture

NP cells were extracted from bovine tails of three different animals and cultured in monolayer (<P3) in standard culture media. Cells were then embedded into 3D alginate beads as described previously.^41^ Briefly, cells were mixed in sodium alginate and then added to CaCl_2_ solution in droplets to polymerize. Beads were cultured in standard media for 2 weeks to allow cells to redifferentiate. After this, beads were dissolved in alginate dissolving buffer (55 mM sodium citrate, 30 mM EDTA, 0.15 M NaCl in H_2_O). However, instead of further digesting the ECM deposited by cells through the collagenase digestion step, this step was omitted in order to retain the pericellular matrix deposited during alginate bead culture. Cells were finally encapsulated in alginate disc constructs as described by Reference [42]. This slow‐polymerizing gel was chosen to allow for stiffer alginate constructs that would retain their shape once cut and enable compressive loading. Cells were added to sodium alginate at approximately 1 × 10^6^ cells/mL. CaCO_3_ was then added at a concentration of 45 mM and mixed by pipette, before adding 90 mM glucono‐δ‐lactone and mixing. This solution was added to 6‐well plates at 6.7 ml/well and allowed to polymerize for 45 min at 37°C before culture media was added. After 24 h incubation, gels were harvested for mechanical stimulation. Gels were cut using a 7 mm biopsy punch, creating constructs of 7 mm in diameter and in height. Four such constructs were mechanically stimulated from cells of each of the three different animal sources.

#### 2.6.2 Dynamic compression

Dynamic compression stimulation was modeled on that performed by Reference [15], whereby alginate constructs were stimulated with dynamic compressive strain alternating between 7 and 13% strain at 1 Hz for 20 h to elucidate a mechanoresponse. For 24 h prior to loading, constructs were cultured in standard culture media with/without 1 mM Nf to inhibit L‐type VGCCs (which includes Ca_v_1.2). Dynamic compression was performed using the Biodynamic 5200 (TA Instruments) at 37°C. Constructs were placed between the loading platens and the chamber filled with fresh culture media with/without Nf and 12.5 mM HEPES to buffer the media. Immediately after a minimal preload of 25 mN was applied to ensure that the platen was in contact with the construct, dynamic compressive strain was applied as described. Free‐swelling constructs in a similar culture media and environment were used as unloaded controls.

#### 2.6.3 Gene expression

Following dynamic compression, constructs were immediately digested in sodium citrate for 10 min and 0.4 mg/mL collagenase type I for a further 10 min. After cells were isolated, RNA was extracted using a RNeasy Mini Kit (Qiagen) following the manufacturer's instructions and cDNA synthesis was performed using reverse transcription mastermix composed of 1.5 μl deoxynucleotide triphosphate (Meridian Bioscience), 1 μl random hexamers (Invitrogen), 5 μl Bioscript 5× Reverse Transcription Buffer (Bioline), 0.5 μl Bioscript Reverse Transcription Enzyme (Bioline) and 28 μl sterile dH_2_O. RT‐qPCR was performed to investigate the differences in gene expression between each loading condition. Several genes were chosen for investigation, all of which were TaqMan assay primer/probe mixes from ThermoFischer Scientific: aggrecan (Bt03212186_m1), collagen 1a1 (Bt03225322_m1), MMP‐3 (Bt04259490_m1), MMP‐13 (Bt03214050), ADAMTS‐4 (Bt03224693_m1) PTHrP (Bt03224327_m1), IL‐1α (Bt03212736_m1) and IL‐1β (Bt03212741_m1), as well as the VGCCs targets of CACNA1B (Bt03215568_m1), and CACNA1C (Bt07106677_g1). Following initial evaluations of GAPDH, 18S and YWHAZ, the two housekeeping genes of GAPDH (Bt03210913_g1) and 18S (Hs99999901_s1) were used as controls as YWHAZ showed poor expression in many samples. Each reaction was performed in duplicate and a no‐template control was included in every plate. RT‐qPCR was performed by the comparative Ct method using the QuantStudio 7 Flex Machine over 50 cycles. Only samples with a maximum range of 2 between measured Ct values of duplicates were included in analysis, or if one measured value is considered an outlier after statistical analysis. Data were transformed to 2^−∆Ct^ values for analysis, as described by Reference [43], and multiplied by a factor of 10^3^ for col 1a, aggrecan, and MMP‐13, a factor of 10^5^ for PTHrP and a factor of 10^6^ for ADAMTS‐4 to present gene expression relationships across treatment conditions more clearly.

### 2.7 Statistical analysis

All statistical data analysis was conducted on SPSS (IBM). Data were first tested for normality using the Shapiro–Wilke test, interpretation of the Q‐Q plot and histogram distribution shape. Differences between groups were analyzed using a One‐way Anova with post‐hoc Tukey HSD tests for normally distributed data, while the Kruskal–Wallis H‐test with post‐hoc Mann–Whitney U‐tests were similarly conducted for non‐parametric data. Two‐tailed and unpaired statistical tests were used with a significance threshold set at 95% confidence (*p* = 0.05). At least three biological replicates were used in all experiments apart from calcium signaling in bovine cells, where such cells were pooled from multiple sources.

## 3 RESULTS

**FIGURE 2:**
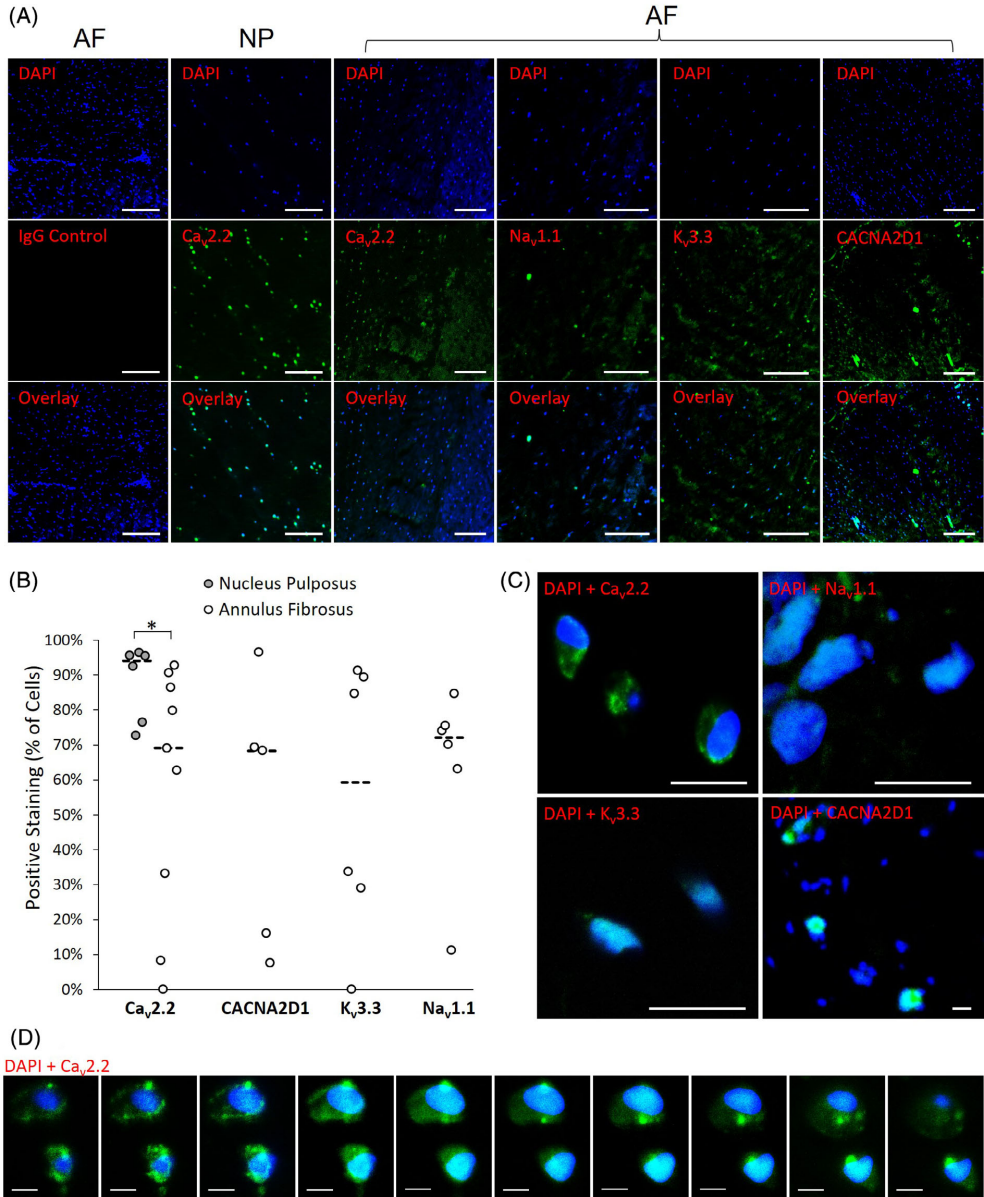
Immunofluorescent staining of voltage‐gated ion channels (VGICs) in bovine nucleus pulposus (NP) and annulus fibrosus (AF) tissue. (A) Representative images of AF and NP tissue stained with DAPI and the channel of interest. All four markers were expressed on AF cells, while only the Ca_v_2.2 channel appeared present on NP cells. Scale bar = 200 μm. (B) Proportion of AF or NP cells that appeared to express the channel of interest. The Ca_v_2.2 channel appeared to be expressed across a significantly greater proportion of NP cells than AF cells (*p* > 0.05), while no difference was found in Ca_v_2.2, Na_v_1.1, K_v_3.3 or CACNA2D1 markers across AF cells. Median values are represented by dashed lines. (C) Localization of the four VGICs at high magnification. The Ca_v_2.2 channel appeared to be expressed throughout the cell, with some localized areas of greater fluorescence. Both the Na_v_1.1 and K_v_3.3 were predominately expressed only at the nucleus, while the CACNA2D1 auxiliary subunit was expressed throughout the cell and at greater fluorescence in areas of cell clusters. Scale bar = 15 μm (D) Z‐stack images of NP cells stained with DAPI and Ca_v_2.2 at 1.5 μm intervals. Scale bar = 5 μm

Fluorescent IHC demonstrated the presence of VGICs in bovine IVD cells (Figure 2). All four VGIC markers of Ca_v_2.2, Na_v_1.1, K_v_3.3 and the auxiliary subunit CACNA2D1 were expressed in bovine AF cells, while only the Ca_v_2.2 channel was expressed in NP cells (Figure 2A). The expression of this channel was significantly higher in NP cells than in AF cells (*p* < 0.05), with 94% of NP cells staining positive for the channel marker compared to 64% of AF cells (Figure 2B). While a range of expression levels were seen across the four VGIC markers in AF cells, no significant difference in expression was found. Higher magnification images allowed for determining the localization of these channels in AF and NP cells and, therefore, inferring their functional roles (Figure 2C,D). The Ca_v_2.2 marker appeared to be expressed throughout the cell, both on the cell membrane as well as through the cell in a punctate staining pattern. This suggests that this channel protein may be stored and transported by vesicles to the cell membrane, or alternatively, localized on the mitochondrial membranes. The Na_v_1.1 and K_v_3.3 markers, on the other hand, appeared to co‐localize with the nucleus, suggesting that these channels function as nuclear membrane VGICs. The CACNA2D1 subunit appeared to be expressed throughout the cell, particularly in areas of cell clusters. Such a widespread expression is to be expected of this auxiliary subunit, which forms several different VGICs. The increased expression among cell clusters, however, suggests a greater role of VGICs in endocrine cell signaling.

**FIGURE 3:**
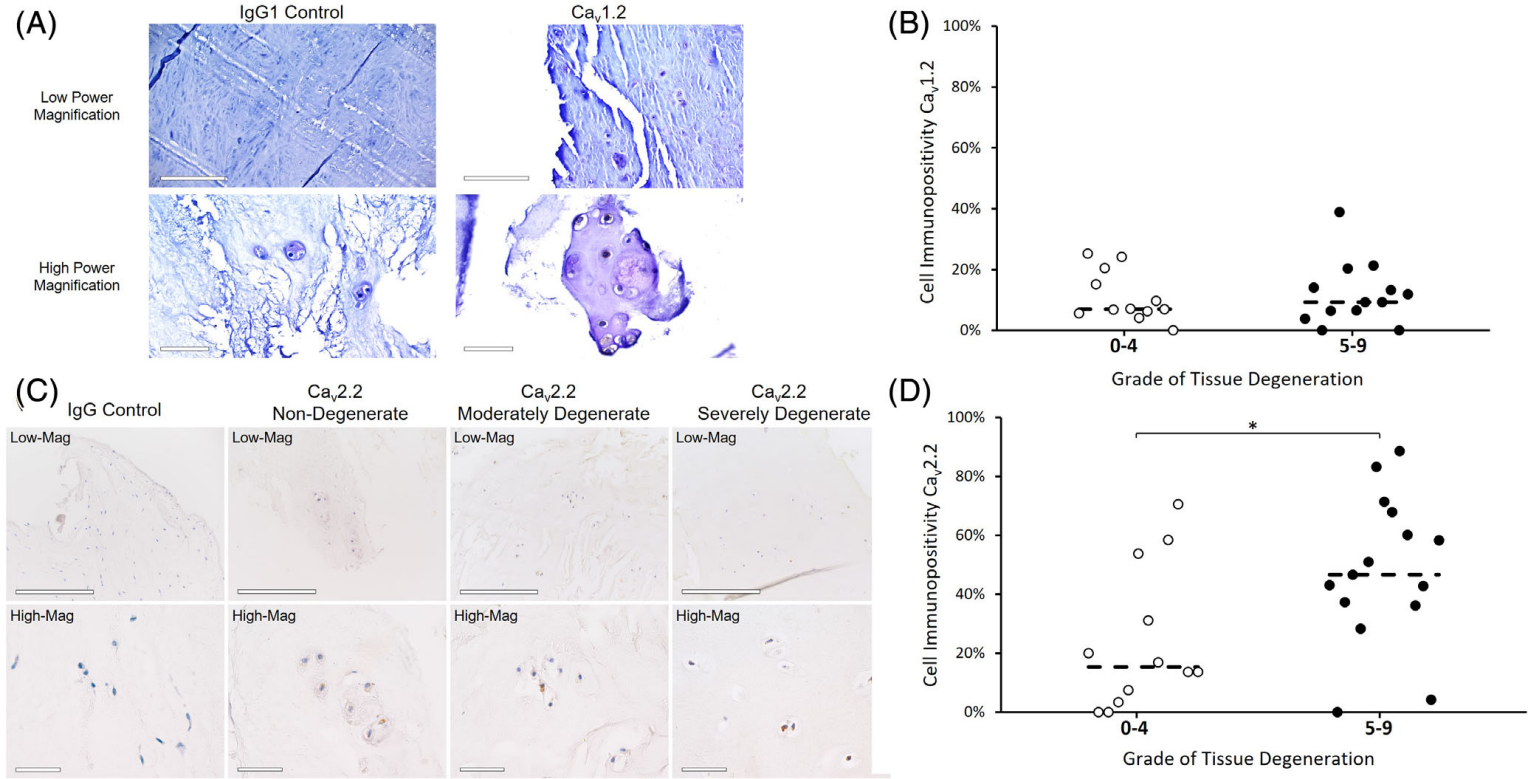
Immunohistochemical (colorimetric) staining of the Ca_v_1.2 and Ca_v_2.2 channel markers on human intervertebral disc (IVD) tissue. (A) Representative images at low and high magnification of human IVD tissue stained for the Ca_v_1.2 marker and DAPI compared to the IgG1 negative control. Scale bar = 200 μm and 50 μm at low and high magnification respectively. (B) The relative proportion of cells that appeared to stain positive for the Ca_v_1.2 in tissue with non/low degeneration (grades 0–4) and medium/severe degeneration (grades 5–9). No statistically significant differences were found in expression levels across degeneration. (C) Representative images at low and high magnification of human IVD tissue stained for the Ca_v_2.2 marker and DAPI across grades of degeneration compared to the IgG negative control. Scale bar = 100 μm and 20 μm at low and high magnification respectively. (D) The relative proportion of cells that appeared to stain positive for the Ca_v_2.2 in tissue with non/low and medium/severe degeneration. There was a significant difference in the proportion of cells that stained positive for Ca_v_2.2 between non/low and medium/severe degenerate tissue sections (*p* < 0.05). Median values are represented by the dashed line

**FIGURE 4:**
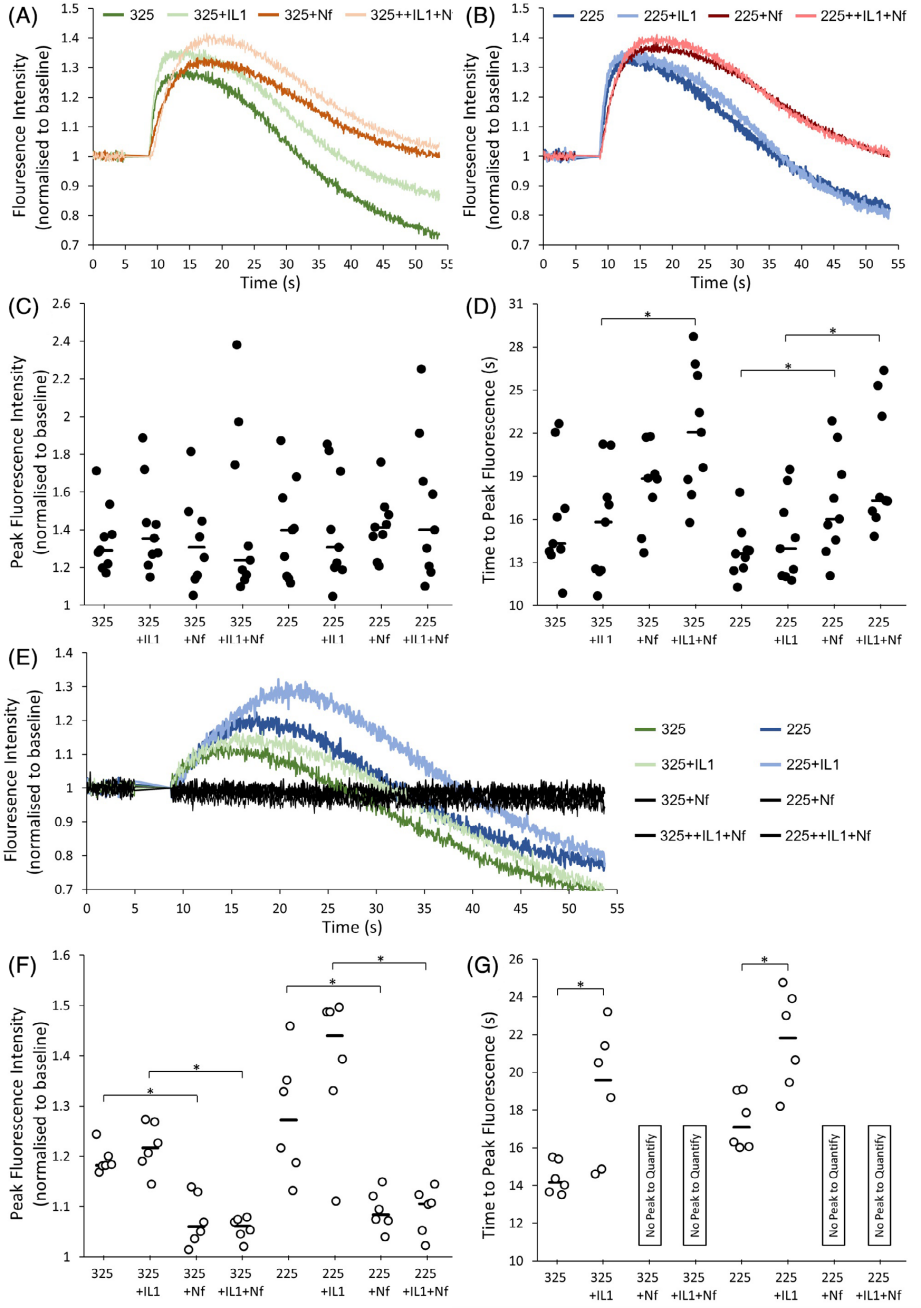
Calcium influx by nucleus pulposus (NP) cells in response to osmotic loading with/without IL‐1 and nifedipine as measured by peak fluorescence intensity after staining with Fluo‐4. (A)–(D) Human NP cells' peak fluorescence intensity and time to reach peak intensity following treatments as labeled, with similar graphs in (E)–(G) for bovine cells. Averaged fluorescence intensity curves for all treatments in human cells are shown for treatments with standard (325 mOsm) media and osmotically altered (225 mOsm) media in (A) and (B) respectively. There was no difference found in the magnitude of peak fluorescence intensity across any treatment (C), while significant differences were found in the time to reach peak intensity (D) through nifedipine supplementation. Median values are represented by horizontal lines while significant differences (*p* < 0.05) are highlighted by the *. Similar averaged fluorescence curves are shown in (E) for bovine cells, with similar plots for peak fluorescence intensities in (F) and (G). As shown, nifedipine supplementation in all treatment groups caused a complete inhibition of calcium influx for bovine cells

When IHC was then performed on human samples, both the Ca_v_1.2 and Ca_v_2.2 channels were identified, with no evidence of the Na_v_1.1, K_v_3.3 or CACNA2D1 markers across any cells, although it should be noted that the majority of human IVD samples investigated consisted mainly of NP tissues (Figure 3). While the Ca_v_1.2 channel was expressed in tissue samples across all grades of degeneration (Figure 3A), no difference was found across degeneration grade, although expression was seen in a low number of cells (Figure 3B). Similar to bovine cells, the Ca_v_2.2 channel was again expressed throughout the cell and in greater intensity at localized areas outside the nucleus, indicating its potential role as a cell membrane VGIC (Figure 3C). In the relative quantification of this marker across grades of disc degeneration, a significant increase in Ca_v_2.2 expression was seen with degeneration (*p* < 0.05) (Figure 3D). Once the presence of VGICs were confirmed at the protein level in the human and bovine IVD, the second part of this study investigated the function of some of these channels. In carrying out a study on VGIC roles in IVD cell mechanotransduction, the L‐type family of VGCCs were selected for study due to their known roles in mediating cartilage mechanoresponses. Firstly, the underlying ability of L‐type VGCCs to affect intracellular signaling was determined by NP cell calcium influx in response to stimulation with/without Nf via Fluo‐4 direct calcium assay (Figure 4). There was no significant difference in the magnitude of fluorescence intensity, or calcium influx, in response to any of the treatment conditions in human cells (Figure 4A,B). Nf supplementation did, however, significantly delay calcium uptake (*p* < 0.05) in human NP cells treated with IL‐1 (Figure 4C, as well as in cells osmotically loaded with 225 mOsm media with/without IL‐1 (Figure 4D). In bovine NP cells, Nf completely inhibited any calcium uptake beyond baseline levels in all treatment. IL‐1 supplementation also resulted in a significant delay in calcium influx, both in standard 325 mOsm media and osmotic loading with 225 mOsm media (Figure 4F), which again was further completely inhibited by Nf.

**FIGURE 5:**
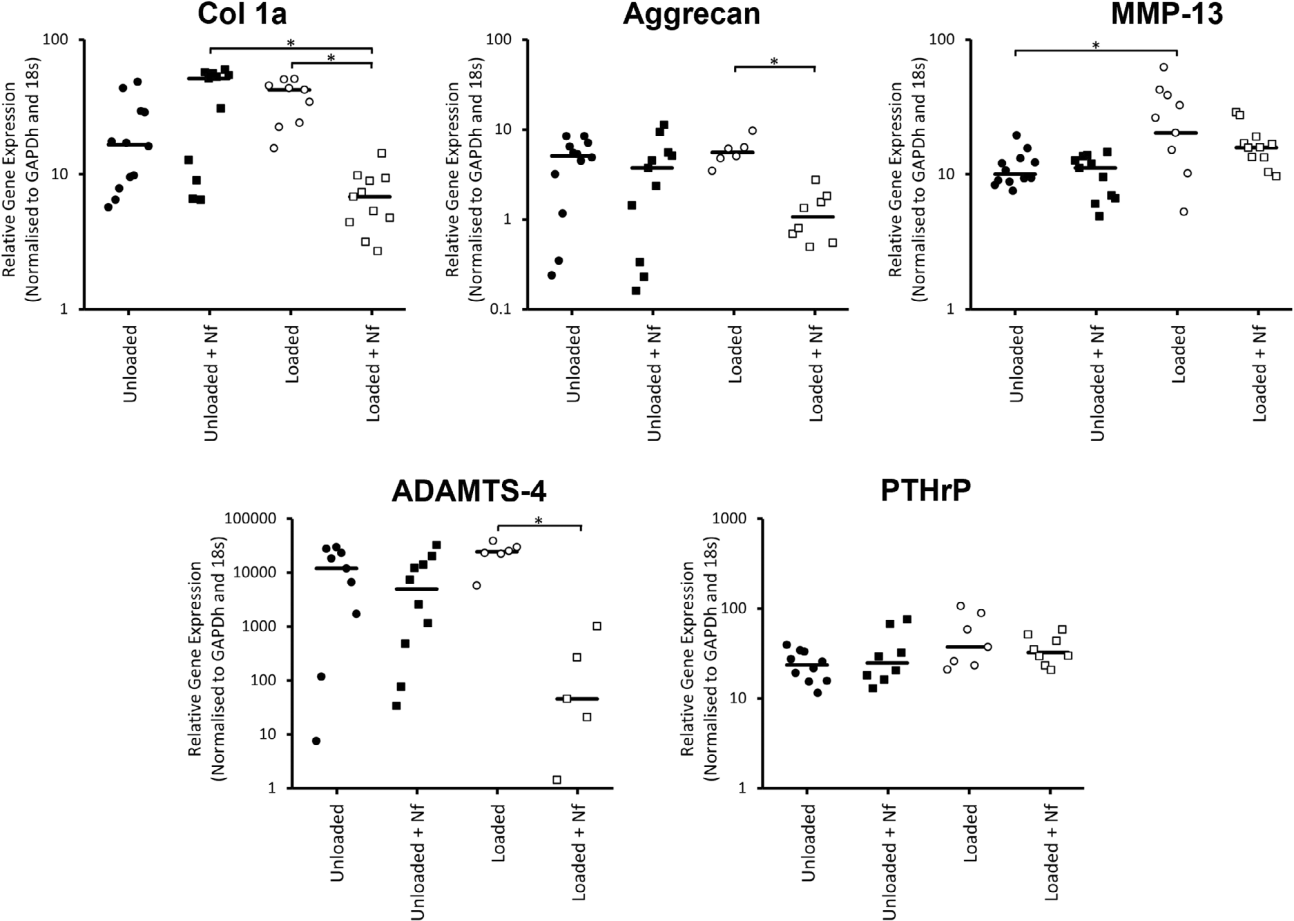
Gene expression of anabolic and catabolic factors from bovine nucleus pulposus cells encapsulated in alginate constructs and subject to different treatment conditions, as shown, of dynamic compression with/without Nf supplementation and unloaded, no‐supplementation controls. While qRT‐PCR was unsuccessful on some samples, at least 12 samples from three biological repeats were tested in each condition. Median values are represented by the horizontal line while significant differences (*p* < 0.05) are represented by the *

Bovine NP cells encapsulated in alginate constructs were also subjected to dynamic compression with/without Nf to inhibit VGCCs. Dynamic compression alone only led to a significant change in one gene tested, an upregulation of the catabolic marker MMP‐13 (Figure 5). However, inhibiting L‐type VGCCs by Nf supplementation in tandem with dynamic compression resulted in significant downregulation of col 1a, aggrecan and ADAMTS‐4 compared to constructs subject to the same compression in standard media, while no difference was seen with Nf treatment in unloaded samples (Figure 5). Specific to col 1a, compression with Nf supplementation also resulted in significant downregulation compared to Nf supplementation in free‐swelling constructs, while no condition caused a change in PTHrP. MMP‐3, IL‐1α, IL‐1β, CACNA1B, and CACNA1C were undetected by qPCR within these bovine NP samples.

## 4 DISCUSSION

The IVD is mechanically loaded during normal daily activities, the cells within the disc respond to these forces through a variety of mechanoreceptors. Within articular cartilage VGICs are known to be an important mechanoreceptor,^15^, ^16^, ^17^ however, to date only murine microarray data has suggested IVD cells express VGICs.^25^ Whilst mechanotransduction pathways in the IVD have been shown to be altered during IVD degeneration, such as integrin mediated response,^20^ to date VGIC expression and function during degeneration has not been investigated. This study has demonstrated, for the first time, the presence of VGIC markers in the IVD at the protein level. Furthermore, staining of human tissue has revealed that the expression of one VGIC, Ca_v_2.2, increases with the onset of degeneration. The localization of Ca_v_2.2 in bovine cells around the cell membrane, in contrast to the localization of Na_v_1.1 and K_v_3.3 around the nuclear membrane, further supports the likely different roles that these VGICs may play in cell signaling and mechanotransduction.

While all four VGIC markers investigated were present in similar relative proportions across bovine AF cells, the expression of Ca_v_2.2 was found to be greater in the NP, a finding supported by genomic data from Reference [25] Ca_v_2.2 is a N‐type voltage‐gated calcium channel, and thereby activated by a high voltage stimulation^44^ while being partly responsible for calcium influx in chondrocytes.^45^ While a high percentage of NP cells expressed the Ca_v_2.2 channel relative to AF cells, in contrast to the lack of Na_v_1.1, K_v_3.3 and CACNA2D1 staining in the NP, many different factors could be responsible for differential expression levels, such as glucose concentration^46^ and pH,^47^ which themselves are different across the IVD.^48^ Additionally, this differential expression may suggest that these channels are involved in the mechanoresponse to forces that are more frequently generated in the NP or AF, such as compression or tension, respectively.

The expression of Ca_v_2.2 across human disc samples, as shown through colorimetric IHC, provides further evidence for this VGIC in the IVD. As the proportion of cells expressing this channel significantly increased with the onset of degeneration, Ca_v_2.2 may be involved in mediating a response to a force that itself is increased with degeneration, such as compression or shear‐stress.^9^ Alternatively, increased Ca_v_2.2 expression may be a result of an increase in calcium ions in the degenerate IVD along with tissue calcification.^31^, ^49^ As IVD cells undergo phenotype changes in degeneration, the altered expression of this VGIC seems to be one such change that may be a result of altered biomechanics and an altered SGP and could lead to changes in mechanotransduction pathways in IVD degeneration. While this study was not able to investigate AF tissue in human IVD sections regarding VGIC quantification, due to a lack of AF tissues from surgical samples, further investigations on the possible differential expression of these VGICs between AF and NP tissue in the human IVD, as done here with bovine tissue, are warranted.

In localizing these VGICs, Ca_v_2.2 was expressed along the cell membrane, indicating its role as a cell membrane ion channel. This suggests that only Ca_v_2.2 may be involved in the cellular calcium flux in response to an electrical potential along the membrane as the other channels investigated did not show membrane staining. Furthermore, punctate staining was observed for Ca_v_2.2 which may indicate vesicular staining or localization on the mitochondrial membrane. Several mitochondrial membrane ion channels have previously been shown to present with a similar punctate staining pattern through the cell,^50^, ^51^ while many VGICs have also presented with punctate staining.^52^ As the mitochondria membrane also maintains a resting membrane potential,^53^ and as mitochondrial calcium flux is an important signaling mechanism in protein synthesis,^54^ Ca_v_2.2 may play a role in IVD cell mitochondrial ion flux. Evidence also shows that mitochondrial ion channels and calcium flux are involved in the cell mechanoresponse,^55^, ^56^, ^57^ indicating a role in IVD cell mechanotransduction, regardless of whether Ca_v_2.2 functions as a cell membrane‐bound or mitochondrial membrane‐bound ion channel.

The localization of Na_v_1.1 and K_v_3.3 around the nucleus, conversely, indicates that these channels may be involved in nuclear membrane transport of intracellular sodium and potassium ions. Although not being localized to a specific VGIC, CACNA2D1, as an auxiliary subunit to many channels, was expressed primarily at cell clusters and throughout the cell within bovine AF cells. VGCCs that possess this sub‐unit may therefore be more involved in close‐proximity endocrine cell signaling, while this localization distinct from the Ca_v_2.2 channel also demonstrates the presence of some unknown VGCCs. A range of calcium‐ and potassium‐specific VGICs have been shown to regulate nuclear membrane potential and the activation of some transcription factors in cancer cells.^58^, ^59^ These channels may work in conjunction with IVD mechanotransduction through downstream gene transcription processes as it has been suggested that potassium‐specific channels may regulate other voltage gated calcium channels along the inner and outer nuclear membrane.^60^

Colorimetric IHC also revealed the expression of Cav1.2, an L‐type VGCCs, across the human IVD but the relative channel expression was not found to correlate with grade of degeneration. As this family of L‐type VGCC are known to be involved in the chondrocyte mechanoresponse to a variety of forces, a pilot study on L‐type VGCC function in the disc was therefore conducted through calcium signaling and mechanical stimulation investigations.

Intracellular calcium oscillations are widely known to be important signaling pathways across a range of cellular processes, such as differentiation and proliferation, with even brief spikes of calcium influx sufficient to trigger a response in non‐excitable cells.^61^, ^62^ Given that chondrocytes mediate calcium flux in part through VGCCs,^37^ intracellular calcium flux was therefore deemed a suitable focus of this study to initially determine the role of VGCCs in IVD cells. Inhibition of L‐type VGCCs in both bovine and human cells caused significant changes in cellular calcium influx when stimulated. The delay in calcium influx caused by Nf supplementation in human cells demonstrates that these VGCCs are involved in mediating the cell response. This is supported by Reference [37], who found that Nf supplementation lead to altered frequency, rather than magnitude, of spontaneous chondrocyte calcium oscillations. In bovine cells, the complete inhibition of calcium influx provides further support to the physiological role of these channels. As hypotonic solution causes membrane depolarization in healthy human chondrocytes but fails to do so in degenerate chondrocytes,^63^ IL‐1 was introduced to model a degenerate environment in monolayer for this study, as done previously.^28^, ^36^ However, IL‐1 supplementation alone caused no observable effect in calcium influx in human cells and had the opposite effect in bovine cells, causing an increase in calcium influx in response to an osmotic and, potentially, fluid‐induced shear stress (by media injection) load. In either loading case, however, Nf supplementation reduced these peaks to comparable negligible levels.

Proceeding to a 3D dynamic compression study and downstream gene analysis yielded further evidence of the role of VGCCs in IVD cell mechanotransduction. The downregulation of both anabolic and catabolic markers in mechanically stimulated cells with inhibition of L‐type VGCCs suggests that these channels are involved in mediating the cell mechanoresponse to compression. In a similar study on chondrocytes in 3D agarose culture,^15^ previously demonstrated that inhibition of L‐type VGCCs in chondrocytes subject to dynamic compression resulted in a significant increase in overall protein synthesis, as measured by radiolabel incorporation, but caused no difference in GAG synthesis. This broadly supports the present study by demonstrating a mechanosensitive role for L‐type VGCCs in a similar cell type, while not directly supporting the gene expression changes observed here. Similarly,^26^ found that Nf supplementation downregulated several genes in chondrocytes subject to dynamic compression. In other forms of mechanical stimulation, L‐type VGCCs were found to mediate chondrocyte proliferation, differentiation, and matrix synthesis in response to cyclic tensile strain.^16^, ^39^ Given that several other VGICs were found to be expressed only in the AF, functional studies of these channels in mediating the AF cell response to physiological loading such as cyclic tensile strain may provide further mechanistic insight into these channels in the IVD. Beyond cell culture studies,^38^ found that inhibiting L‐type VGCCs in an explant model of chick embryo hindlimbs subject to dynamic compression resulted in reduction in joint growth to similar levels seen in static culture.

While the findings of this pilot mechanotransduction study suggests that L‐type VGCCs are involved in mediating the expression of several genes in response to mechanical stimulation, these come with many limitations and caveats. Firstly, downstream analysis was only conducted at the gene level and may not reflect changes at the protein level. Secondly, while the methods of this study were closely modeled on more thorough studies, various magnitudes, frequencies, and end points of dynamic compression, as well as Nf concentrations, were not investigated. Thirdly, bovine cells were the topic of the study and, as such, resultant gene expression changes may not translate to that of human cells taken from a generally more degenerate environment. However, while the present study serves as an initial investigation regards the involvement of L‐type VGCCs in IVD cell mechanotransduction, the evidence for such a role in chondrocytes through cell and explant studies supports these preliminary findings and justifies further detailed studies on VGICs in the IVD.

## 5 CONCLUSION

Mechanotransduction is known to be partly mediated by VGICs in chondrocytes, while, until now, these channels had yet to be elucidated in the IVD. Several different VGICs have been identified across the bovine and human IVD, with expression levels altered by disc region, localization across the cell and grade of degeneration in the human disc. Through functional investigations, L‐type VGCCs were found to play a role in the NP cell response to osmotic stimulation, mediating both the magnitude of calcium influx and the rate at which this response occurred. 3D dynamic compression studies further highlighted the role these channels play in mediating the mechanoresponse of NP cells to physiological loading, with altered gene expression of several factors when inhibiting L‐type VGCCs. These findings underline the importance of further study of VGICs in the IVD, particularly with regards to mechanotransduction. Only through fully elucidating the presence and function of VGICs in the disc, as well as other potential signaling pathways, can strategies for tackling IVD degeneration be fully informed and better outcomes for patients suffering from LBP be achieved.

## AUTHOR CONTRIBUTIONS

All authors contributed to the design of the study. Joseph W. Snuggs and Christine L. Le Maitre performed grading of the IVD samples. Philip Poillot performed the IHC staining and calcium flux experiments. Philip Poillot and Joseph W. Snuggs performed the mechanotransduction study including 3D culture and gene analysis. All authors contributed to the analysis of the data and preparation of the manuscript. All authors approved the final submitted manuscript.

## CONFLICT OF INTEREST

The authors declare that they have no known competing financial interests or personal relationships that could have appeared to influence the work reported in this paper.
